# The association between blood vitamin E and blood pressure in an adult population with and without diabetes mellitus

**DOI:** 10.3389/fendo.2024.1431293

**Published:** 2024-11-06

**Authors:** Rong Wan, Yuhao Su, Meilan Zhu, Ying Huang

**Affiliations:** ^1^ Jiangxi Key Laboratory of Molecular Medicine, The Second Affiliated Hospital, Jiangxi Medical College, Nanchang University, Nanchang, Jiangxi, China; ^2^ Department of Cardiovascular Medicine, The Second Affiliated Hospital, Jiangxi Medical College, Nanchang University, Nanchang, Jiangxi, China; ^3^ Rehabilitation Department, The Second Affiliated Hospital, Jiangxi Medical College, Nanchang University, Nanchang, Jiangxi, China

**Keywords:** alpha-tocopherol, gamma-tocopherol, blood pressure, diabetes, linear regression

## Abstract

**Aims:**

Previous studies on the association between vitamin E and blood pressure (BP) levels are controversial. Our study aimed to evaluate the association between blood vitamin E (alpha-tocopherol and gamma-tocopherol) levels and systolic and diastolic BP in an adult population with diabetes and without diabetes.

**Methods:**

Our study data were obtained from a biomarker project of the Midlife in the United States (MIDUS) study. A total of 1068 subjects were included, and the associations between alpha-tocopherol and gamma-tocopherol levels and systolic and diastolic BP were further analyzed by smooth curve and multivariate linear regression analyses.

**Results:**

Our smooth curve analysis showed an almost linear correlation between blood vitamin E (alpha-tocopherol and gamma-tocopherol) levels and systolic and diastolic BP. Furthermore, we found that blood gamma-tocopherol levels were positively and independently associated with systolic BP (B=0.427, 95% CI 0.067-0.787, P=0.020) and diastolic BP (B=0.289, 95% CI 0.072-0.507, P=0.009) when the data were adjusted for age, gender, body mass index (BMI), ever smoked cigarettes regularly, number of years of consuming alcohol and regular exercise or activity for 20 minutes or more at least 3 times/week. Consistently, blood alpha-tocopherol levels were also positively associated with systolic BP (B=0.150, 95% CI 0.064-0.235, P=0.001) and diastolic BP (B=0.056, 95% CI 0.004-0.107, P=0.035) after these variables were adjusted. However, these significant relationships exist only in subjects without diabetes, but not in subjects with diabetes.

**Conclusions:**

We observed for the first time that blood vitamin E (alpha-tocopherol and gamma-tocopherol) levels were positively associated with systolic and diastolic BP in subjects without diabetes.

## Introduction

Hypertension has been considered a global, common, important public health problem (1, 2). Existing evidence reported that approximately 9.4 million people died of hypertension and its complications, such as coronary heart disease, heart failure and stroke, on a worldwide scale (3), and the hypertensive population might exceed 500 million in the next decade (4). Although many etiologies or risk factors, including aging, obesity, a high-salt diet, less exercise, high social pressure and others for hypertension, have been identified, we are unable to do anything about these risk factors or the intervention is not sufficiently effective (5). Therefore, early prevention of hypertension has important clinical significance for the prevention of cardiovascular disease (CVD). Nitric oxide (NO) mainly originated from renal tubular cells and vascular endothelium plays an important role in keeping blood pressure (BP) within a normal range (6). Previous studies have suggested that reduced NO expression contributes to the development of hypertension (7). For example, one study reported increased expression of reactive oxygen species (ROS), elevated oxidative stress and a low inflammatory response in hypertensive subjects, which can reduce NO bioavailability (8). NO bioavailability can be regulated and controlled by excessive ROS production in hypertensive patients (9). Importantly, the cellular production of antioxidants can also promote NO expression due to the beneficial properties of antioxidants in scavenging free radicals. Antioxidants play vital roles in reducing ROS production and increasing NO bioavailability (10).

As an antioxidant, vitamin E is a type of fat soluble vitamin that includes four tocopherols and four tocotrienols. All vitamin E forms also have relatively consistent antioxidant properties (11). Existing studies have demonstrated that vitamin E can exert protective effects on endothelial function in the vasculature by scavenging ROS and exerting anti-inflammatory properties, which can further promote NO expression and bioavailability (11, 12). One study also showed that compared with placebo, vitamin E supplementation for approximately 7 months could lower BP in mild hypertensive subjects (13). Moreover, some clinical trials have suggested the beneficial impacts of vitamin E supplementation on BP in different populations (14, 15).

However, few studies have investigated the association between blood vitamin E levels and BP in the adult population with diabetes. We conducted a cross-sectional analysis to investigate the association between blood vitamin E (alpha-tocopherol and gamma-tocopherol) levels and BP in the adult population with diabetes and without diabetes.

## Methods

Our study data were obtained from the Midlife in the United States (MIDUS) study, involving more than seven thousand nonhospitalized adults aged from 25 to 75 years. The MIDUS study started in 1995 (16), and a 9-year follow-up (MIDUS 2) was conducted between 2004 and 2006 with 4,963 subjects (17). A subgroup of these subjects participated in a biomarker study (N = 1255) (18). Each subject was invited to undergo a comprehensive examination at clinical research centers. The comprehensive physical examination, biological sample collection and medical history records were conducted by trained medical staff. Full details of the MIDUS study biomarker protocol are available elsewhere (17–19). In the biomarker study, fasting blood samples were obtained from patients before breakfast, and then these samples were sent to the MIDUS Biocore laboratory for further analysis. In the present study, blood levels of gamma-tocopherol, alpha-tocopherol, hemoglobin A1c %, fasting glucose, fasting insulin, total cholesterol, triglycerides, high-density lipoprotein cholesterol (HDL-C) and low-density lipoprotein cholesterol (LDL-C) were analyzed. A history of diabetes was registered as “diagnosed with diabetes” or “without diabetes” in this dataset. In summary, after excluding subjects with missing data (N=187) in the biomarker study, the remaining 1068 subjects were included and further analyzed in our cross-sectional analysis. Accordance to the Declaration of Helsinki, all protocols in present study were granted full ethical approval as part of the MIDUS 2 Biomarker Project and informed consent was obtained from each participant.

### Confounding variables

The following variables were included as confounding variables at the biomarker clinic visit: age, sex (male and female), body mass index (BMI), smoking (ever smoked cigarettes regularly), drinking (number of years of alcohol consumption) and physical activity (regular exercise for 20 minutes or more at least 3 times/week or not). BMI is defined as weight (kg) divided by the square of height (m^2^).

### Statistical analysis

All of the data were analyzed by using SPSS 26.0 and EmpowerStats. *P* ≤ 0.05 was considered to indicate a statistically significant results. For the purpose of the study, all included study samples were divided into two groups (diagnosed with diabetes and without diabetes) according to the history of diabetes. These continuous variables were analyzed using the Mann–Whitney U test, and categorical variables were analyzed by the chi-square test. Then, a smooth curve was performed to evaluate the associations between blood vitamin E (alpha-tocopherol and gamma-tocopherol) levels and systolic and diastolic BP. Furthermore, multivariate linear regression models were used to evaluate the associations of blood alpha-tocopherol and gamma-tocopherol levels and BP by adjusting for confounding factors. Confounding factors, including age and sex, were included in Model 1. Adjustment of confounding factors, including age, sex and BMI, was performed in Model 2. In Model 3, adjustments for age, sex, BMI, ever smoked cigarettes regularly and number of years consuming alcohol were performed. In Model 4, adjustments for age, sex, BMI, ever smoked cigarettes regularly, number of years consuming alcohol were and regular exercise for 20 minutes or more at least 3 times/week were performed. Finally, to investigate whether diabetes has an impact on the relationships between blood vitamin E (alpha-tocopherol and gamma-tocopherol) levels and systolic and diastolic BP, stratified analysis was performed by adding “ever diagnosed with diabetes” as the stratification variable.

## Results

The demographic characteristics and lifestyles of all included participants are described in **Table 1**. The median age of participants was 53 years, and 487 participants were male (45.6%). The median values of blood gamma-tocopherol and alpha-tocopherol levels were 3.04 µmol/L and 26.06 µmol/L, respectively. The median systolic BP and diastolic BP values were 130 mmHg and 75 mmHg, respectively. These participants were divided into two groups (diagnosed with diabetes and without diabetes). Participants diagnosed with diabetes tended to have a higher age and lower rate of physical activities and had higher levels of blood gamma-tocopherol and alpha-tocopherol than participants never diagnosed with diabetes (all P <0.05). However, differences in systolic and diastolic BP were not noted between the group diagnosed with diabetes and the group without diabetes.

**Table 1:** Characteristics of participants (N=1068).

Variables	All participants (N=1068)	With diabetes (N=123)	Without diabetes (N=945)	P value
Age (year)	53 (45-62)	56 (48-64)	53 (44-62)	<0.001
Gender (male), n (%)	487 (45.6)	61 (49.6)	426 (45.1)	0.387
BMI (kg/m^2^)	28.5 (25.1-32.8)	31.83 (28.52-36.18)	28.0 (25.0-32.4)	0.467
Ever smoked cigarettes regularly, n (%)	546 (51.1)	68 (55.3)	478 (50.6)	0.339
Number of years drank that much (years)	5 (2-15)	5 (2-20)	5 (2-15)	0.166
Regular exercise for 20 minutes or more at least 3 times/week, n (%)	823 (77.1)	85 (69.1)	738 (78.1)	0.030
Average systolic BP (mmHg)	130 (118-143)	134 (121-144)	130 (118-143)	0.809
Average diastolic BP (mmHg)	75 (68-82)	73 (67-82)	75 (69-83)	0.622
Diabetes, n (%)	123 (11.5)	–	–	–
Blood markers				
Blood gamma-tocopherol (umol/L)	3.04 (1.88-4.95)	3.51 (2.03-5.97)	2.99 (1.86-4.87)	0.022
Blood alpha-tocopherol (umol/L)	26.06 (20.11-34.17)	26.09 (18.62-32.63)	26.02 (20.22-34.22)	0.002
Blood hemoglobin A1c %	5.82 (5.60- 6.20)	7.20 (6.50-9.03)	5.74 (5.50-6.04)	0.016
Blood fasting glucose levels (mg/dL)	96.0 (90.0-104.0)	120.0 (104.0-154.0)	95.0 (89.0-102.0)	0.063
Blood fasting insulin levels (uIU/mL)	10.0 (6.0-16.0)	15.0 (10.0-25.0)	9.0 (6.0-15.0)	0.474
Blood total cholesterol (mg/dl)	185.0 (160.0-212.0)	165.0 (149.0-187.0)	187.0 (162.0-214.0)	0.183
Blood triglycerides (mg/dl)	106.0 (76.0-154.8)	126.0 (90.0-187.0)	105.0 (75.0-150.5)	0.412
Blood HDL-C (mg/dl)	53.0 (43.0-66.0)	48.0 (40.0-64.0)	54.0 (43.0-67.0)	0.784
Blood LDL-C (mg/dl)	102.0 (81.0-128.0)	86.0 (67.0-109.0)	104.0 (83.0-130.0)	0.164

BMI, body mass index; BP, blood pressure; HDL-C, high-density lipoprotein cholesterol; LDL-C, low-density lipoprotein cholesterol.

**Figure 1:**
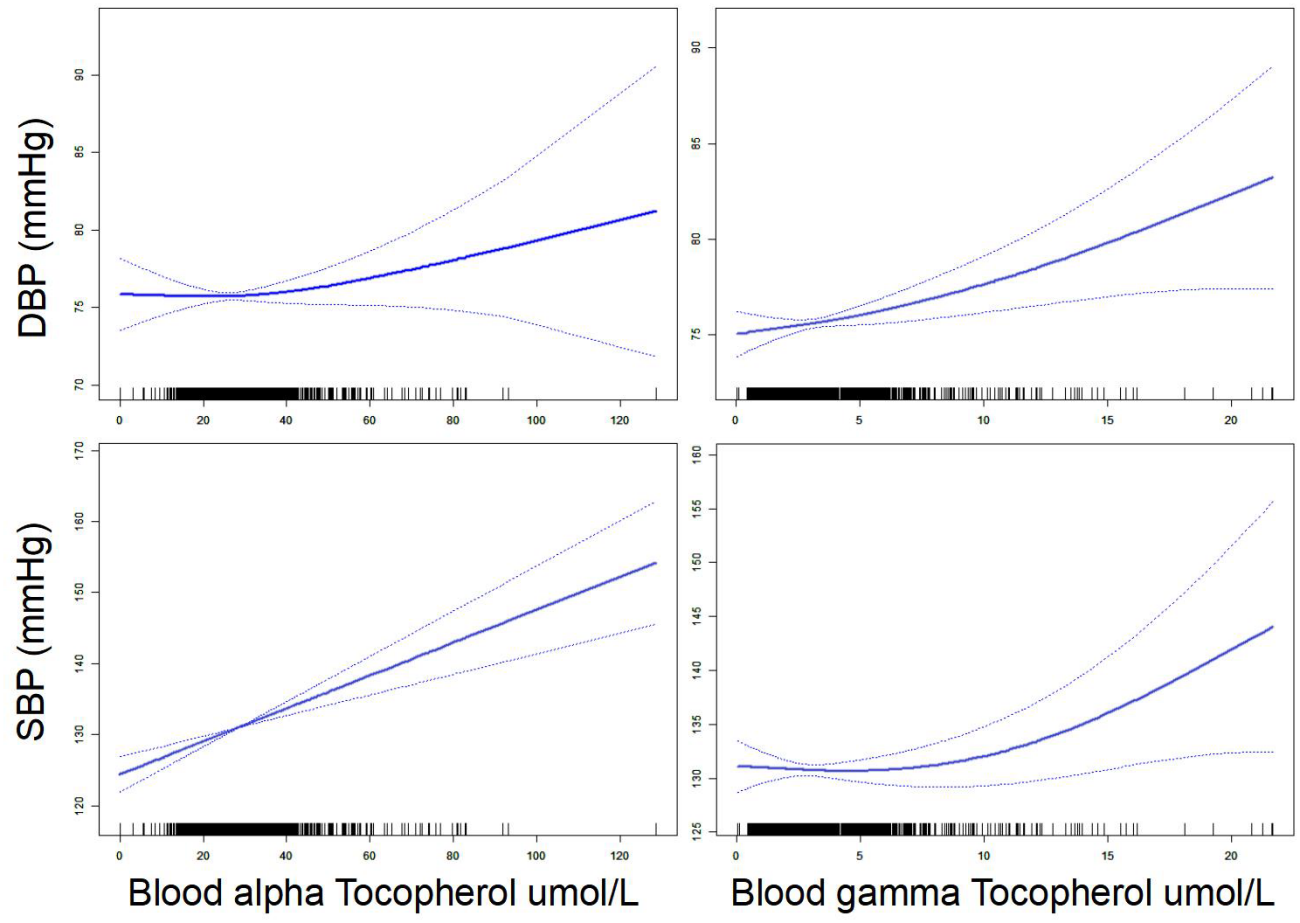
Association between vitamin E (alpha-tocopherol and gamma-tocopherol) and blood pressure.

First, our smooth curve analysis showed an almost linear correlation between blood vitamin E (alpha-tocopherol and gamma-tocopherol) levels and systolic and diastolic BP (**Figure 1**). For further clarity of the association between blood vitamin E (alpha-tocopherol and gamma-tocopherol) levels and BP, multivariate linear regression analysis was used. Our results suggested that blood gamma-tocopherol levels were positively related to systolic BP (B=0.519, 95% CI 0.156-0.883, P=0.005) and diastolic BP (B=0.300, 95% CI 0.084-0.515, P=0.006) when age and gender were adjusted in Model 1 (**Table 2**). Similarly, blood alpha-tocopherol levels were positively related to systolic BP (B=0.161, 95% CI 0.075-0.246, P<0.001) and diastolic BP (B=0.059, 95% CI 0.007-0.110, P=0.025) after adjusting for age and gender in Model 1. Furthermore, these positive associations between blood vitamin E (alpha-tocopherol and gamma-tocopherol) levels and systolic and diastolic BP changed slightly after adjusting for BMI, ever smoked cigarettes regularly and number of years of alcohol consumption in Models 2 and 3. Finally, we found that blood gamma-tocopherol levels were still positively associated with systolic BP (B=0.427, 95% CI 0.067-0.787, P=0.020) and diastolic BP (B=0.289, 95% CI 0.072-0.507, P=0.009) when further adjusting for regular exercise or activity for 20 minutes or more at least 3 times/week in Model 4. Consistently, blood alpha-tocopherol levels were also positively associated with systolic BP (B=0.150, 95% CI 0.064-0.235, P=0.001) and diastolic BP (B=0.056, 95% CI 0.004-0.107, P=0.035).

**Table 2:** Relationship between blood gamma-tocopherol and alpha-tocopherol levels and BP.

Variables	Systolic BP			Diastolic BP		
	B 95% CI	Sβ	*P*	B 95% CI	Sβ	*P*
Gamma-tocopherol (umol/L)						
**Model 1**	0.519 (0.156-0.883)	0.083	0.005	0.300 (0.084-0.515)	0.082	0.006
**Model 2**	0.399 (0.040-0.758)	0.064	0.029	0.280 (0.064-0.497)	0.076	0.011
**Model 3**	0.421 (0.062-0.781)	0.067	0.022	0.291 (0.074-0.508)	0.079	0.009
**Model 4**	0.427 (0.067-0.787)	0.068	0.020	0.289 (0.072-0.507)	0.079	0.009
Alpha-tocopherol (umol/L)						
**Model 1**	0.161 (0.075-0.246)	0.110	<0.001	0.059 (0.007-0.110)	0.068	0.025
**Model 2**	0.157 (0.073-0.241)	0.108	<0.001	0.058 (0.007-0.109)	0.068	0.026
**Model 3**	0.151 (0.066-0.236)	0.103	0.001	0.055 (0.003-0.106)	0.064	0.038
**Model 4**	0.150 (0.064-0.235)	0.103	0.001	0.056 (0.004-0.107)	0.065	0.035

Model 1: Adjusted for age and gender.

Model 2: Adjusted for age, gender and BMI.

Model3: Adjusted for age, gender, BMI, ever smoked cigarettes regularly and number of years drank that much.

Model 4:Adjusted for age, gender, BMI, ever smoked cigarettes regularly, number of years drank that much and regular exercise or activity for 20 minutes or more at least 3 times/week.

To assess whether diabetes has an impact on this relationship between blood vitamin E (alpha-tocopherol and gamma-tocopherol) levels and systolic and diastolic BP, stratified analysis by adding “diagnosed with diabetes” as the hierarchical variable was performed. As shown in **Table 3**, blood gamma-tocopherol levels were significantly associated with systolic BP (B=0.446, 95% CI 0.044-0.849, P=0.030) and diastolic BP (B=0.317, 95% CI 0.075-0.560, P=0.010) in subjects without diabetes after adjusting for age, gender, BMI, ever smoked cigarettes regularly, number of years consuming alcohol and regular exercise or activity for 20 minutes or more at least 3 times/week in Model 4. Similarly, blood alpha-tocopherol levels were still significantly associated with systolic BP (B=0.149, 95% CI 0.057-0.241, P=0.002) and diastolic BP (B=0.062, 95% CI 0.007-0.118, P=0.028) in subjects without diabetes after adjusting for the same confounding factors in Model 4.

**Table 3:** Relationship between blood gamma-tocopherol and alpha-tocopherol levels and BP in patients without diabetes (N=945).

Variables	Systolic BP			Diastolic BP		
	B 95% CI	Sβ	*P*	B 95% CI	Sβ	*P*
Gamma-tocopherol (umol/L)						
**Model 1**	0.546 (0.138-0.954)	0.083	0.009	0.338 (0.097-0.579)	0.087	0.006
**Model 2**	0.425 (0.025-0.825)	0.064	0.037	0.306 (0.065-0.547)	0.079	0.013
**Model 3**	0.441 (0.039-0.844)	0.067	0.032	0.318 (0.075-0.560)	0.082	0.010
**Model 4**	0.446 (0.044-0.849)	0.068	0.030	0.317 (0.075-0.560)	0.082	0.010
Alpha-tocopherol (umol/L)						
**Model 1**	0.163 (0.070-0.257)	0.110	0.001	0.066 (0.011-0.121)	0.076	0.019
**Model 2**	0.153 (0.062-0.244)	0.102	0.001	0.063 (0.008-0.118)	0.072	0.025
**Model 3**	0.151 (0.059-0.243)	0.101	0.001	0.061 (0.006-0.117)	0.070	0.030
**Model 4**	0.149 (0.057-0.241)	0.100	0.002	0.062 (0.007-0.118)	0.071	0.028

Model 1: Adjusted for age and gender.

Model 2: Adjusted for age, gender and BMI.

Model3: Adjusted for age, gender, BMI, ever smoked cigarettes regularly and number of years drank that much.

Model 4:Adjusted for age, gender, BMI, ever smoked cigarettes regularly, number of years drank that much and regular exercise or activity for 20 minutes or more at least 3 times/week.

However, inconsistently, blood gamma-tocopherol levels were not related to systolic BP (B=0.315, 95% CI -0.564-1.195, P=0.479) or diastolic BP (B=0.230, 95% CI -0.285-0.745, P=0.378) in subjects with diabetes after adjustments were made in Model 4 (**Table 4**). Additionally, blood alpha-tocopherol levels were not related to systolic BP (B=0.092, 95% CI -0.140-0.325, P=0.433) or diastolic BP (B=-0.012, 95% CI -0.149-0.124, P=0.858) in subjects with diabetes after adjusting for the same confounding factors in Model 4.

**Table 4:** Relationship between blood gamma-tocopherol and alpha-tocopherol levels and BP in patients with diabetes (N=123).

Variables	Systolic BP			Diastolic BP		
	B 95% CI	Sβ	*P*	B 95% CI	Sβ	*P*
Gamma-tocopherol (umol/L)						
**Model 1**	0.419 (-0.460-1.297)	0.087	0.348	0.280 (-0.235-0.795)	0.097	0.284
**Model 2**	0.409 (-0.475-1.292)	0.085	0.361	0.302 (-0.210-0.815)	0.105	0.245
**Model 3**	0.348 (-0.522-1.219)	0.072	0.430	0.282 (-0.232-0.797)	0.098	0.280
**Model 4**	0.315 (-0.564-1.195)	0.065	0.479	0.230 (-0.285-0.745)	0.080	0.378
Alpha-tocopherol (umol/L)						
**Model 1**	0.161 (-0.065-0.386)	0.129	0.161	0.030 (-0.104-0.163)	0.040	0.662
**Model 2**	0.164 (-0.062-0.390)	0.132	0.154	0.024 (-0.109-0.157)	0.032	0.772
**Model 3**	0.100 (-0.130-0.331)	0.081	0.391	0.001 (-0.136-0.138)	0.001	0.989
**Model 4**	0.092 (-0.140-0.325)	0.074	0.433	-0.012 (-0.149-0.124)	-0.017	0.858

Model 1: Adjusted for age and gender.

Model 2: Adjusted for age, gender and BMI.

Model3: Adjusted for age, gender, BMI, ever smoked cigarettes regularly and number of years drank that much.

Model 4:Adjusted for age, gender, BMI, ever smoked cigarettes regularly, number of years drank that much and regular exercise or activity for 20 minutes or more at least 3 times/week.

## Discussion

Our study first identified blood vitamin E (alpha-tocopherol and gamma-tocopherol) levels as independently associated with BP in subjects without diabetes but not in subjects with diabetes. The association was consistent in our regression models adjusted for a great range of confounding factors, including demographic characteristics and lifestyle. One of the important explanations for the effect of vitamin E in BP is the NO system (19). NO originating from the vascular endothelium plays a vital role in regulating BP, and endothelial dysfunction caused by various pathological factors subsequently increases BP (20). A systemic excessive accumulation of ROS caused by oxidative stress can eliminate NO and lower NO bioavailability (6). Vitamin E, as a lipid-soluble vitamin, has many important effects on cell protection and the immune system (12). Vitamin E can attenuate oxidative stress and neutralize ROS and thus can improve NO production and bioavailability (12).

In fact, the effect of vitamin E on BP has been investigated in some studies, and these conclusions were inconsistent. For example, previous studies have reported that vitamin E supplementation of 200 IU/day could lead to a greater than 20% reduction in systolic BP over 7 months in hypertensive patients (13). However, a clinical trial demonstrated that vitamin E supplementation of 400 IU daily for 8 weeks did not lower BP levels in a middle-aged and elderly population (21). In view of this finding, a recent meta-analysis of the association between vitamin E supplementation and BP was performed (22). The meta-analysis included 839 participants from 18 clinical trials and reported that supplementation with vitamin E can significantly decrease systolic BP but not diastolic BP (22). Inconsistent with our results, increased blood alpha-tocopherol and gamma-tocopherol levels were associated with elevated systolic and diastolic BP in subjects without diabetes but not in subjects with diabetes. These inconsistent results may account for the inclusion of sample populations with different ages, lifestyles, dietary habits, concomitant diseases, various blood measurements and data analysis methods. Consistent with our results, another clinical trial reported no change in diastolic BP after alpha-tocopherol supplementation of 1200 IU/day for 8 weeks in patients with type 2 diabetes mellitus (T2DM) (23). Additionally, some clinical studies also showed that vitamin E supplementation had no impact on systolic BP in hypertensive subjects (24). However, these studies did not measure blood vitamin E levels, and only oral intake of vitamin E was evaluated, which may be one of the important reasons for the inconsistent results.

Importantly, recent research also implied that vitamin E may have a negative impact on health. For example, an analysis from a cross-sectional study in Germany suggested that increased serum levels of vitamin E were related to a higher content of visceral fat and an increased risk of metabolic syndrome (25), which is similar to our results. However, another study found an inverse association between vitamin E consumption and metabolic syndrome and subsequently showed mild improvement in insulin resistance after three months (26). But, oral intake of vitamin E was assessed in this study instead of its blood levels. Additionally, retinol binding protein (RBP) plays a vital role in maintaining serum levels of vitamin A. Elevated serum RBP levels are associated with higher BP, serum triglyceride levels, BMI and insulin resistance (27, 28). A basic experiment showed similar results that the number of RBP4 variants was related to a higher risk of hypertriglyceridemia (29). Previous studies surrounding oral intake and serum levels of vitamins have also discovered conflicting results in a Korean population. In a study including 404 old adults in Korea, Kim et al. observed an inverse association between oral and serum vitamin (retinol and α-tocopherol) levels and the risk of metabolic syndrome in elderly women (30). Similarly, increased intake of vitamin E was related to a reduced prevalence of metabolic syndrome (31). However, Cho et al. reported that high serum α-tocopherol levels contributed to an increased risk of metabolic syndrome (32), which is consistent with our results.

Some potential mechanisms regarding the negative impacts of antioxidants on CVD health have been proposed. Also, several plausible explanations for vitamin E exist. First, the relationship between serum alpha-tocopherol levels and adipose tissue may affect this association. The content of visceral adipose tissue was positively related to the alpha-tocopherol/cholesterol ratio, which was associated with a higher odds ratio for metabolic syndrome (25). Second, the metabolite levels of tocopherol in blood and urine samples were significantly reduced in patients with metabolic syndrome compared with the healthy population, suggesting that tocopherol catabolism is significantly retarded in these subjects with metabolic syndrome (33). These results seem to partly explain the positive association between vitamin E and BP in subjects without diabetes.

Our study has several notable strengths. On the one hand, we are the first to report that blood vitamin E (alpha-tocopherol and gamma-tocopherol) levels were positively associated with BP in subjects without diabetes but not in subjects with diabetes after adjusting for a great range of confounding factors, including demographic characteristics and lifestyle. On the other hand, our study included a large sample of data from MIDUS, and the analysis was performed by professional researchers. Furthermore, we directly evaluated serum alpha-tocopherol and gamma-tocopherol levels, which reflect the real relationship between vitamin E within the human bod and BP, rather than the oral intake of vitamin E. This information expands data presented in the few relevant studies. Certainly, this study also has several limitations. First, although various confounding factors were adjusted in the study given that these factors may influence blood vitamin E levels, some confounding factors, such as medications and other relevant clinical data that should also be evaluated as confounding variables, cannot be fully eliminated. Second, serum levels of vitamin E were measured only at a single period of time, which might cause survivorship biases in the cross-sectional design. Third, our study lacked data on the postmenopausal status and it may affect the results obtained. Fourth, using many various covariates in our regression analysis may cause overfitting of the model, leading to bias in the results. Fifth, given that this study is a *post hoc* analysis, biases cannot be excluded. Finally, we did not further investigate the mechanisms underlying the association of blood vitamin E with BP.

In conclusion, we revealed that blood vitamin E levels were positively associated with BP in subjects without diabetes rather than those with diabetes. These results are inconsistent with the traditional understanding that antioxidants can improve BP.

## Data Availability

The raw data supporting the conclusions of this article will be made available by the authors, without undue reservation.
